# Relationship between root formation progress of adjacent teeth and external root resorption associated with impacted maxillary canines: a retrospective cross-sectional CBCT study

**DOI:** 10.1186/s40510-026-00638-x

**Published:** 2026-07-14

**Authors:** Yukari Mizoguchi, Yuji Ishida, Aiko Ishizaki-Terauchi, Chiyo Shimizu-Tomoda, Takashi Ono

**Affiliations:** 1 Department of Orthodontic Science, Graduate School of Medical and Dental Sciences, Institute of Science Tokyo (SCIENCE TOKYO), Tokyo, Japan; 2 Center of Excellence in Precision Medicine and Digital Health, Department of Physiology, Faculty of Dentistry, Chulalongkorn University, Bangkok, Thailand

## Abstract

**Background:**

This study aimed to clarify the involvement of the root formation stage and apical foramen diameter (AFD) of adjacent teeth in the occurrence of external root resorption (ERR) associated with impacted maxillary canines (IMCs), and to identify novel risk indicators for more accurate clinical prediction.

**Methods:**

Forty patients with IMCs (58 canines) who underwent cone-beam computed tomography (CBCT) between May 2017 and October 2022 were retrospectively analyzed. The developmental status was assessed using the root formation stage and AFD of the central incisor (U1), lateral incisor (U2), and first premolar (U4). Additionally, spatial factors, including inter-root distance and the 3D position of the IMC, were evaluated in relation to ERR.

**Results:**

ERR was detected in 72.5% of patients, affecting 37.1% of all examined adjacent teeth. Among the affected teeth, U2 showed the highest incidence (65.3%), followed by U1 (28.6%) and U4 (6.1%). In exploratory tooth-level comparisons, AFD was smaller in the ERR group for U1 and U4. In the supplementary affected-side-level mixed-effects analysis, no statistically significant independent association was demonstrated between maximum AFD and side-level ERR occurrence.

**Conclusions:**

Developmental indicators, including AFD, may provide clinically relevant information when assessing ERR associated with IMCs. These indicators should be interpreted as potential modifying factors in conjunction with established spatial relationships rather than as independent predictive markers.

## Introduction

The prevalence of external root resorption (ERR) in cases of an impacted maxillary canine (IMC) has been reported to be approximately 10–15% when assessed using conventional two-dimensional imaging [1]. Studies employing panoramic radiographs have highlighted several limitations of this modality, including image distortion, reduced accuracy in root length measurement, and structural overlap [2] .

**Fig. 1:**
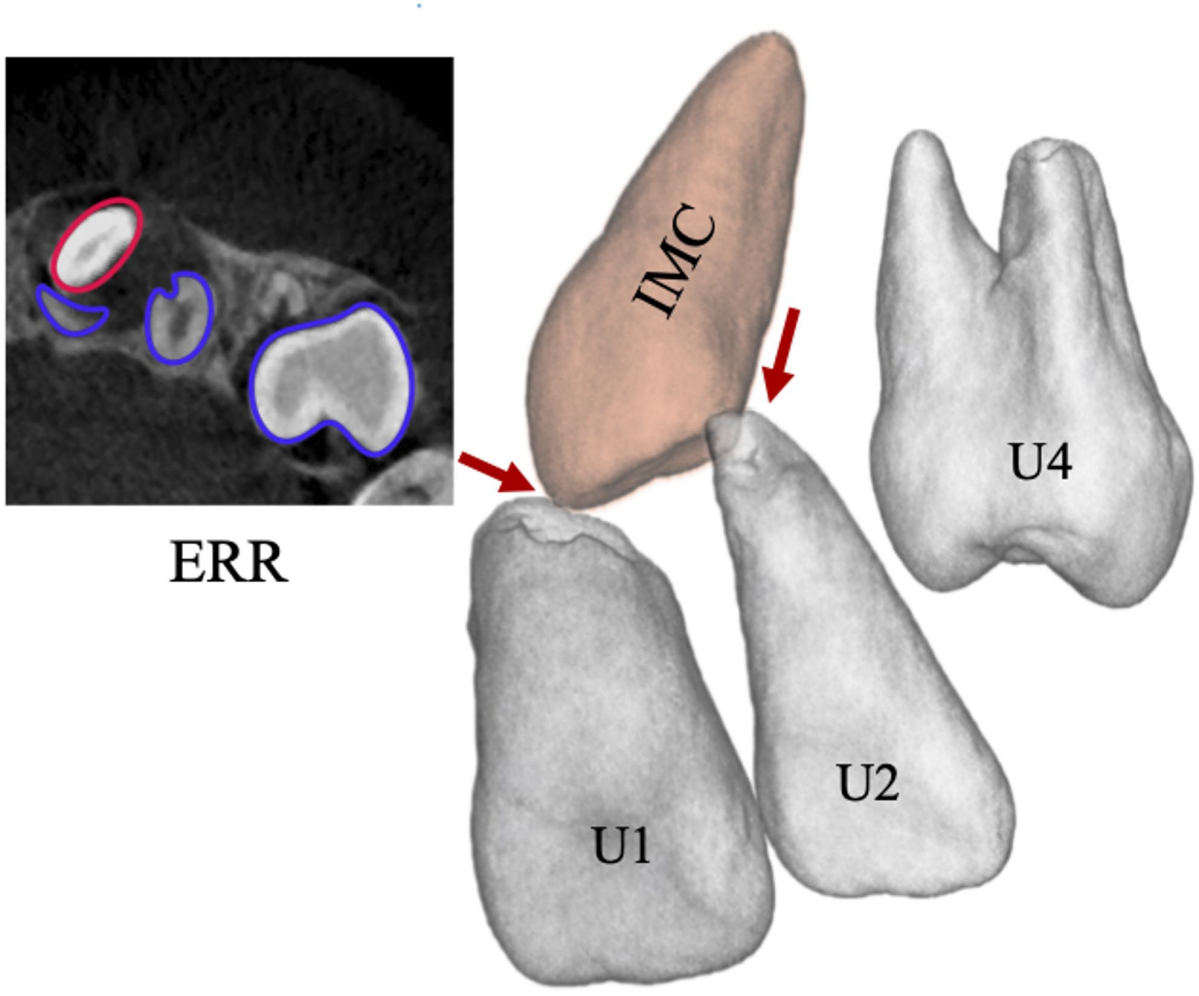
Comparison of developmental and spatial variables according to the presence of external root resorption. A, Root formation stage; B, AFD; C, U2-U4 distance * : p< 0.05, ** : < 0.01. Abbreviations: ERR, external root resorption; AFD, apical foramen diameter; U1, maxillary central incisor; U2, maxillary lateral incisor; U4, maxillary first premolar

Cone-beam computed tomography (CBCT) enables accurate three-dimensional (3D) evaluation of dental structures with reduced measurement error and has been shown to improve the detection of ERR compared with conventional two-dimensional (2D) imaging, particularly in cases involving IMC, where the detection rate of ERR has been reported to improve to 34.6–48.4% [1, 3, 4] (Fig. 1).

Systematic reviews of ERR due to IMC have demonstrated that most of the risk factors of ERR are related to the spatial relationship between the IMC and adjacent teeth [5]. Specifically, factors reported to be associated with the occurrence of ERR include the proximity to or direct contact with adjacent tooth roots, horizontal and vertical positions of the canine cusp, its angulation toward adjacent teeth, and the presence of palatal impaction. Conversely, several factors have been found not to be associated with ERR, including laterality [6], maxillary arch length, sex, age, morphology and size of the adjacent teeth [7], and cyst-like radiolucency surrounding the canine crown [8].

In cases of IMC, the canine germ is often positioned high and mesially within the maxillary alveolar bone, resulting in a longer and more complex eruption path and potentially exerting sustained mechanical pressure on the roots of adjacent teeth, which may induce inflammatory responses leading to ERR in adjacent teeth [9].

In contrast, in cases of severe crowding, although there is insufficient space for the eruption of all teeth, the crowns are typically tilted or rotated, and individual teeth generally do not deviate significantly from their normal positions, with the crowns erupting into the oral cavity, while the roots remain within the alveolar bone [10]. Therefore, owing to the lack of prolonged direct contact between adjacent roots, sustained mechanical pressure is unlikely to occur, and the risk of ERR is considered low [11, 12]. Moreover, during the early stages of root development associated with permanent tooth replacement, mutual ERR appears to be rare even when multiple developing tooth germs are in proximity to or in contact with each other [13]. Conversely, ERR in adjacent teeth is frequently observed in IMC cases, suggesting that the stage of root formation may play a pivotal role in the occurrence of ERR associated with IMC. However, developmental and growth-related factors have not yet been sufficiently elucidated, and the optimal timing of clinical intervention to prevent ERR in adjacent teeth remains controversial. In particular, the relationship between the root formation stage of adjacent teeth and the occurrence of ERR has not been systematically investigated using 3D imaging.

### Specific objectives or hypotheses

Based on this concept, we hypothesized that developmental factors, including the stage of root formation of the adjacent teeth, may be associated with the occurrence of ERR in patients with IMCs. Therefore, this retrospective study aimed to investigate the relationship between adjacent-tooth developmental status and ERR using pretreatment CBCT images of patients with IMCs. These assessments may contribute to more nuanced clinical risk assessment when interpreted together with spatial findings.

## Materials and methods

### Study design

This was a single-center retrospective cross-sectional CBCT study. The study design was evaluated with reference to previously proposed criteria for assessing the risk of bias in studies of external root resorption [14], including participant selection, measurement methods, and reliability assessment.

### Participants, eligibility criteria, and setting

This retrospective study included 98 Japanese patients who were selected from a total of 2187 Japanese patients who visited the hospital between May 2017 and October 2022, and who were diagnosed with unilateral or bilateral IMC during pretreatment screening, in whom CBCT imaging was conducted only when further detailed assessment was considered necessary for the evaluation of IMC and their relationship with adjacent teeth. All imaging procedures were performed in accordance with the ALARA principle to minimize radiation exposure. Patients were excluded if they had a history of orthodontic treatment, trauma to the maxillary anterior teeth, abnormal tooth number, craniofacial anomalies, or syndromes. Additionally, teeth that could not be clearly evaluated on the images due to limitations such as the field of view (FOV) were excluded from the analysis. As a result, the final sample comprised 40 Japanese patients aged 9–18 years [mean (SD): 12.03 (1.56) years; 13 males and 27 females] with a total of 58 IMCs. Due to the retrospective and exploratory nature of this study, no prior sample size determination was conducted; instead, all eligible samples were targeted for analysis. The study was approved by the institutional ethical review board, and all procedures adhered to the Declaration of Helsinki.

CBCT images were obtained using a standardized protocol (3DX FPD8; J. Morita, Kyoto, Japan), with imaging setting of 120 kV, 200 mA, and a voxel size of 0.25 mm. The FOV was adjusted according to the region of interest. Image analysis was performed using dedicated software (OsiriX, Pixmeo SARL, Geneva, Switzerland).

### Exposures, predictors, potential confounders, and effect modifiers

The main developmental variables of interest were the root formation stage and apical foramen diameter (AFD) of the adjacent teeth. Spatial variables included the distance between U2 and U4, the mesiodistal position of the IMC cusp, and the buccolingual position of the IMC. Sex, unilateral or bilateral impaction, tooth type, and patient-level clustering were considered in the descriptive and exploratory analyses as applicable.

The following variables were assessed: the presence or absence of ERR, the root formation stage and AFD of adjacent teeth, distance between U2 and U4, mesiodistal position of the IMC cusp and buccolingual position of the IMC. All evaluations were conducted by a trained orthodontist with experience in CBCT image interpretation. To assess intra-examiner reliability, measurements for all evaluation items were repeated three times by the same examiner at different time points. The reproducibility of the measurements was evaluated using the intraclass correlation coefficients (ICC). The ICC values were 0.97 (95% CI:0.95–0.99) for root formation stage, 0.95 (95%CI: 0.88–0.98) for the AFD, and 0.98 (95% CI:0.96–0.99) for the distance between U2 and U4 (cervical and apical levels), indicating excellent reliability.

**Fig. 2:**
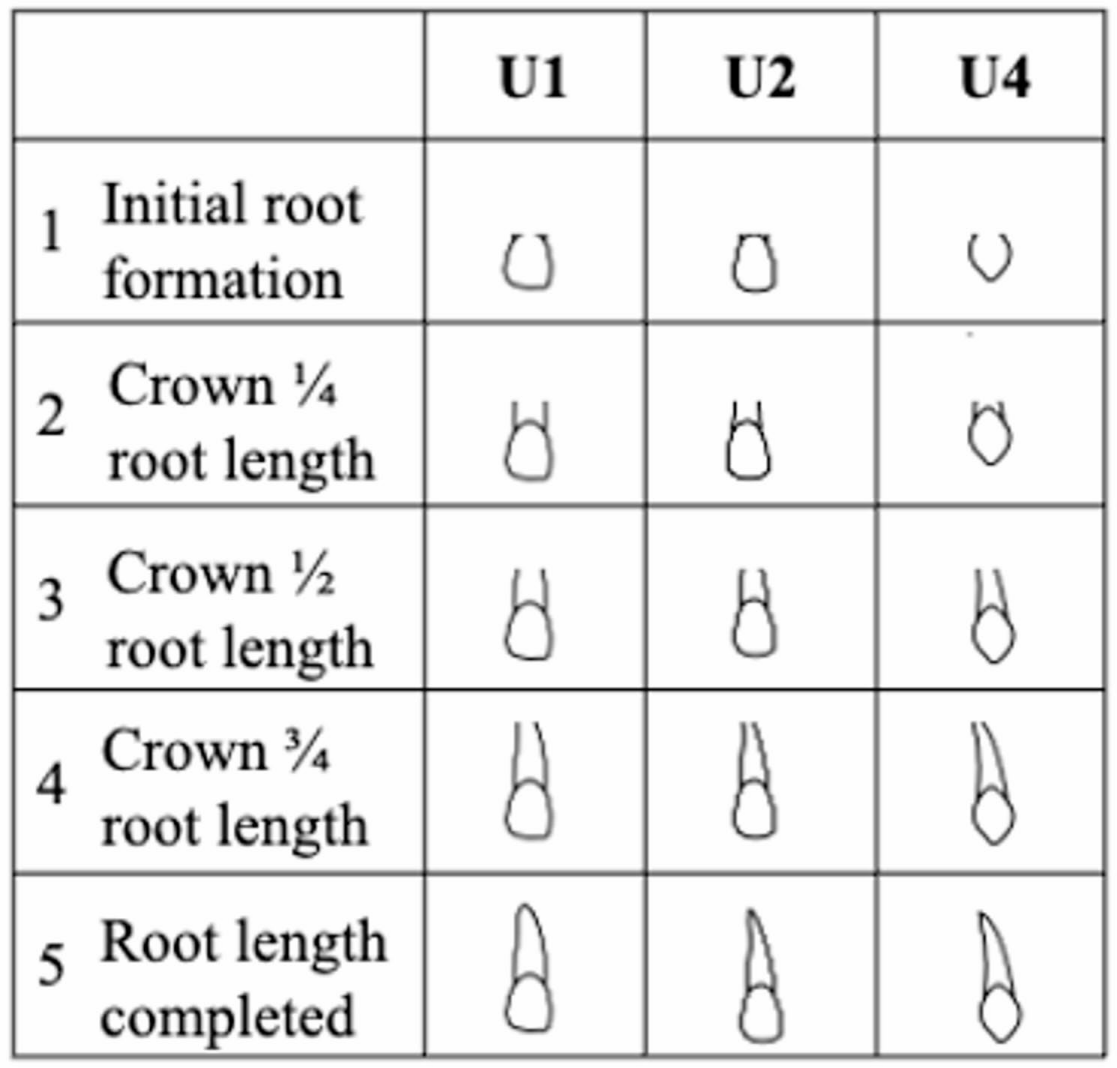
Root formation stages of U1–U4 based on the modified Moorrees classification. Modified from Moorrees et al. (16). Abbreviations: U1, maxillary central incisor; U2, maxillary lateral incisor; U4, maxillary first premolar

Root formation stage: The root formation stages of U1, U2 and U4 on the impacted side were classified into five developmental stages according to a modified version of Moorrees’ classification [15] (Fig. 2).

Apical foramen diameter (AFD): Using axial CBCT sections, the smallest diameter (mm) of the radiographic apical foramen, measured in both the mesiodistal and buccolingual directions, was recorded for U1, U2 and U4.

Distance between U2 and U4: The shortest linear distance (mm) between the root surfaces of U2 and U4 was measured using axial CBCT slices. For standardization, the CBCT images were reoriented via multiplanar reconstruction, using the palatal plane as the reference plane. To account for vertical discrepancies between the two teeth, the distance was measured horizontally, parallel to the palatal plane. The cervical level was defined as the slice corresponding to the cement-enamel junction (CEJ) at the distal aspect of U2 and the mesial aspect of U4. Similarly, the apical level was defined based on the respective root apices of U2 and U4. The U2-U4 distance was selected not as a substitute for direct canine-root distance, but as a measure of the anatomical eruption corridor between adjacent roots. Direct canine-root distance is clinically intuitive and is an established spatial predictor; however, in a retrospective cross-sectional dataset it may partly reflect the current contact or resorption status, with values approaching zero once contact or ERR is present. In contrast, U2-U4 distance describes the available inter-root space surrounding the expected eruption path and was therefore evaluated as a complementary spatial indicator.

**Fig. 3:**
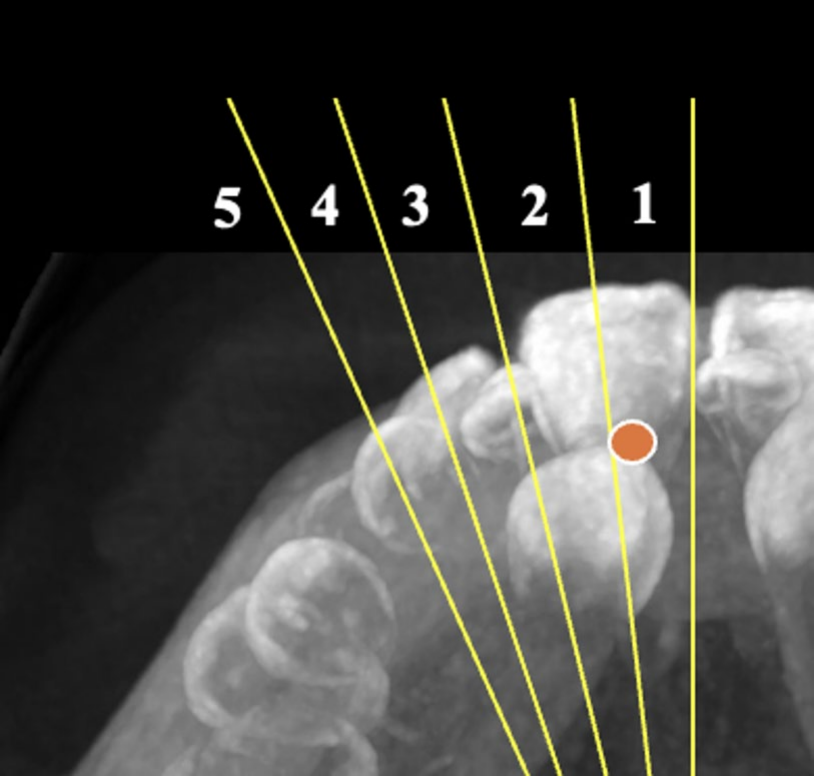
Mesiodistal position of IMC in sector 1–5. The red circle indicates the cusp tip of the IMC. The red circle represents the cusp tip of the IMC located in Sector 1–5. The sectors are categorized as follows: 1, mesial half of the central incisor; 2, distal half of the central incisor; 3, mesial half of the lateral incisor; 4, distal half of the lateral incisor; 5, distal to the lateral incisor.Abbreviation: IMC, impacted maxillary canine

Mesiodistal position of the IMC cusp: Based on the 3D MIP axial images, the cusp position of the IMC was classified into five sectors according to previously described methods [16] (Fig. 3).

Buccolingual position of the IMC: Using axial CBCT images, the position of the IMC was categorized as buccal or palatal. A reference line connecting the buccal and palatal surfaces of the adjacent teeth was drawn to determine the buccolingual position. When more than half of the IMC crown was located outside this line, the corresponding side (buccal or palatal) was designated as the direction of impaction [17]. In cases where the crown intersected the reference line, the impaction direction was determined based on the side where the majority of the crown was situated.

### Outcomes (primary and secondary)

ERR: Identified on CBCT images as the presence of a discontinuity or defect of the root surface. The assessment was performed using axial and multiplanar reconstructed images. For teeth with completed root formation, ERR was defined on the presence of discontinuity or defect of the root surface [18]. For developing teeth, mesiodistal and buccolingual cross-sectional planes were established along the long axis of the tooth and ERR was defined as the presence of a discontinuity or defect observed along the lateral surface of the root when traced from the most apical extent of root formation. Horizontal cross-sectional images perpendicular to the long axis were evaluated by moving from the cervical region toward the apex, and cases in which discontinuity or defect was observed on the external surface of the root were identified as ERR [14]. Cases meeting any of the above criteria were classified as ERR. Based on the evaluation, subjects were classified into ERR and non-ERR groups according to the presence or absence of ERR in the adjacent teeth associated with IMC.

The primary outcome was the presence or absence of ERR on the affected side, defined as ERR in any ipsilateral adjacent tooth (U1, U2, or U4). Secondary outcomes included tooth-level ERR status and the distribution of developmental and spatial variables according to ERR status.

### Sample size calculation

No a priori sample size calculation was performed because of the retrospective and exploratory nature of the study. All eligible patients who met the inclusion criteria during the study period were included.

### Sources of bias and comparability between exposure groups

Potential sources of bias included selection bias related to the retrospective single-center design and the clinical indication for CBCT imaging, measurement bias in the assessment of developing roots, and outcome-related missingness of developmental measurements in teeth with severe apical resorption. To reduce measurement bias, all CBCT assessments were performed using a standardized protocol by a trained orthodontist, and intra-examiner reliability was assessed as described above.

### Statistical analysis (primary and secondary outcomes, subgroup analyses, confounding missing data)

Because this study was exploratory rather than predictive, the main analyses focused on comparing affected sides with and without ERR and on describing tooth-level distributions. Root formation stage, AFD, and U2-U4 distance were evaluated graphically. Because the data appeared non-normally distributed, exploratory tooth-level comparisons between groups were performed using the Mann-Whitney U test (two-sided). Associations between buccolingual position (buccal vs. palatal) and ERR were evaluated using 2 × 2 contingency tables. Odds ratios (ORs) with 95% confidence intervals (CIs) were calculated, and Fisher’s exact test was used. To account for multiple comparisons, p-values were adjusted using the Holm method. All statistical analyses were performed using Prism (version 9.0; GraphPad Software, San Diego, CA, USA).

In addition, a tooth-level mixed-effects logistic regression model was considered. However, this approach was not selected as the primary analysis because severe ERR involving the apical region may obscure or destroy the anatomical landmarks required to evaluate the original root formation stage and AFD. Excluding such teeth from a tooth-level model would preferentially remove clinically important severe cases and could introduce outcome-related selection bias. Therefore, affected-side-level analyses were used to compare sides with and without ERR while reducing the risk of excluding severe ERR cases.

To account for clustering of affected sides within the same patient, exploratory supplementary mixed-effects logistic regression models were fitted with patient ID as a random intercept. Each affected side was treated as one analytical unit; unilateral cases contributed to one affected side, and bilateral cases contributed to two affected sides. The non-affected contralateral side was not included because it was not considered to be exposed to the local effect of the IMC. Side-level ERR occurrence was defined as the presence of ERR in any ipsilateral adjacent tooth, including U1, U2, or U4. For each affected side, the minimum root formation stage and maximum AFD among U1, U2, and U4 were calculated. Minimum root formation stage and maximum AFD were evaluated in separate models because these variables reflect related but distinct aspects of root development. These models were considered supplementary because of the limited sample size and the potential for outcome-related missingness in developmental variables.

## Results

### Participant flow (include flow diagram, early stopping, and time periods)

Among 2,187 Japanese patients who visited the hospital between May 2017 and October 2022, 98 patients were diagnosed with unilateral or bilateral IMC during pretreatment screening. After applying the eligibility criteria and excluding teeth that could not be clearly evaluated on CBCT images, the final sample comprised 40 patients with 58 IMCs. No early stopping was applicable because this was a retrospective study.

### Baseline data (include baseline table)

Baseline characteristics are summarized in Table 1. A total of 58 IMCs were identified: 34 on the right side (58.6%) and 24 on the left side (41.4%). Unilateral impaction was observed in 22 patients (55.0%), whereas bilateral impaction was observed in 18 patients (45.0%). ERR was observed in 29 patients (72.5%), while 11 patients (27.5%) showed no evidence of ERR. Among patients with ERR, 14 (48.3%) exhibited unilateral involvement and 15 (51.7%) exhibited bilateral involvement.

**Table 1:** Baseline characteristics of the study population

Characteristic	Value
Patients, n	40
Age, mean (SD), years	12.03 (1.56)
Sex, n (%)	
Male	13 (32.5)
Female	27 (67.5)
Type of impaction, n (%)	
Unilateral	22 (55.0)
Bilateral	18 (45.0)
Impacted maxillary canines, n	58
Affected side of impacted canine, n (%)	
Right	34 (58.6)
Left	24 (41.4)
Contact with adjacent roots, n (%)	
Yes	29 (72.5)
No	11 (27.5)
Patients with ERR, n (%)	
Yes	29 (72.5)
No	11 (27.5)
ERR distribution among patients with ERR, n (%)	
Unilateral ERR	14 (48.3)
Bilateral ERR	15 (51.7)

Percentages for sex, type of impaction, and patients with ERR were calculated using the number of patients as the denominator (*n* = 40). Percentages for affected side were calculated using the number of impacted maxillary canines as the denominator (*n* = 58). Percentages for ERR distribution were calculated using the number of patients with ERR as the denominator (*n* = 29). Values are presented as mean (SD) or n (%)

Abbreviations: ERR, external root resorption; SD, standard deviation

### Numbers analyzed for each outcome, estimation and precision (include table with inferential statistics results)

A total of 49 teeth exhibited ERR. Among these, U2 showed the highest frequency with 32 teeth (65.3%), followed by U1 with 14 teeth (28.6%) and U4 with 3 teeth (6.1%). When calculated based on the number of IMCs (*n* = 58), the incidence of ERR was 55.2% in U2, 24.1% in U1, and 5.2% in U4 (Table 2).

**Table 2:** Distribution of external root resorption according to tooth type and sex A. Tooth-level distribution of ERR by adjacent tooth type

Tooth type	Teeth with ERR, *n*	Proportion among ERR teeth, %	Incidence per impacted canine, %
U1	14	28.6	24.1
U2	32	65.3	55.2
U4	3	6.1	5.2
Total	49	100	84.5

“Proportion among ERR teeth” was calculated using the total number of teeth with ERR as the denominator (*n* = 49). “Incidence per impacted canine” was calculated using the total number of impacted maxillary canines as the denominator (*n* = 58)

**B. Patient-level association between sex and ERR**

Regarding sex differences, ERR was observed in 11 of 13 males (84.6%) and 18 of 27 females (66.7%). Although the frequency of ERR was higher in males, the difference was not statistically significant (OR = 2.75, 95% CI: 0.50-15.16; Fisher’s exact test, *p* = 0.286) (Table 2).

Regarding the mesiodistal position of the IMC cusp, ERR tended to occur in the adjacent tooth located closest to the sector of the IMC cusp. U1 ERR was mainly observed in sectors 1 and 2, U2 ERR in sectors 2–4, and U4 ERR only in sector 5 (Table 3).

**Table 3:** Distribution of external root resorption by mesiodistal sector of impacted maxillary canine cusp

Sector of IMC cusp	U1 ERR/ total, *n* (%)	U2 ERR/ total, *n* (%)	U4 ERR/ total, *n* (%)
Sector 1	4/5 (80.0)	3/5 (60.0)	0/5 (0.0)
Sector 2	7/13 (53.8)	9/13 (69.2)	0/13 (0.0)
Sector 3	3/19 (15.8)	14/19 (73.7)	0/19 (0.0)
Sector 4	0/10 (0.0)	6/10 (60.0)	0/10 (0.0)
Sector 5	0/11 (0.0)	0/11 (0.0)	3/11 (27.3)

Values are shown as the number of teeth with ERR divided by the total number of evaluable teeth in each sector, with percentages in parentheses. Sector 1 indicates the mesial half of the central incisor; sector 2, the distal half of the central incisor; sector 3, the mesial half of the lateral incisor; sector 4, the distal half of the lateral incisor; and sector 5, distal to the lateral incisor. Only adjacent teeth that could be clearly identified and assessed on CBCT images were included in the analysis

Abbreviations: ERR, external root resorption; IMC, impacted maxillary canine; U1, maxillary central incisor; U2, maxillary lateral incisor; U4, maxillary first premolar

With respect to the buccolingual position of the IMC, 48 IMCs (82.8%) were buccally impacted and 10 (17.2%) were palatally impacted. Palatal impaction was used as the reference category. No significant association was observed between buccolingual position and ERR in U1 or U2. The OR for U4 was not calculated because no ERR events were observed in the palatal group (Table 4).

**Table 4:** Association between buccolingual position of impacted maxillary canines and external root resorption in adjacent teeth

Tooth	Buccolingual position of IMC	ERR, *n* (%)	Non-ERR, *n* (%)	OR	95% CI	*p*-value
U1	Palatal	3(21.4)	7(15.9)	1.00	Reference	
	Buccal	11(78.6)	37(84.1)	0.53	0.13–2.17	0.381
U2	Palatal	6(18.7)	4(15.3)	1.00	Reference	-
	Buccal	26(81.3)	22(84.7)	0.95	0.25–3.54	0.935
U4	Palatal	0(0.0)	10(18.2)	-	-	-
	Buccal	3(100.0)	45(81.8)	-	-	-

Palatal impaction was used as the reference category for odds ratio calculation. Odds ratios indicate the odds of ERR in buccally impacted canines compared with palatally impacted canines. The OR for U4 was not calculated because no ERR events were observed in the palatal group. Percentages were calculated within each ERR status for each tooth type. P-values were calculated using Fisher’s exact test

Abbreviations: CI, confidence interval; ERR, external root resorption; IMC, impacted maxillary canine; OR, odds ratio; U1, maxillary central incisor; U2, maxillary lateral incisor; U4, maxillary first premolar

**Fig. 4:**
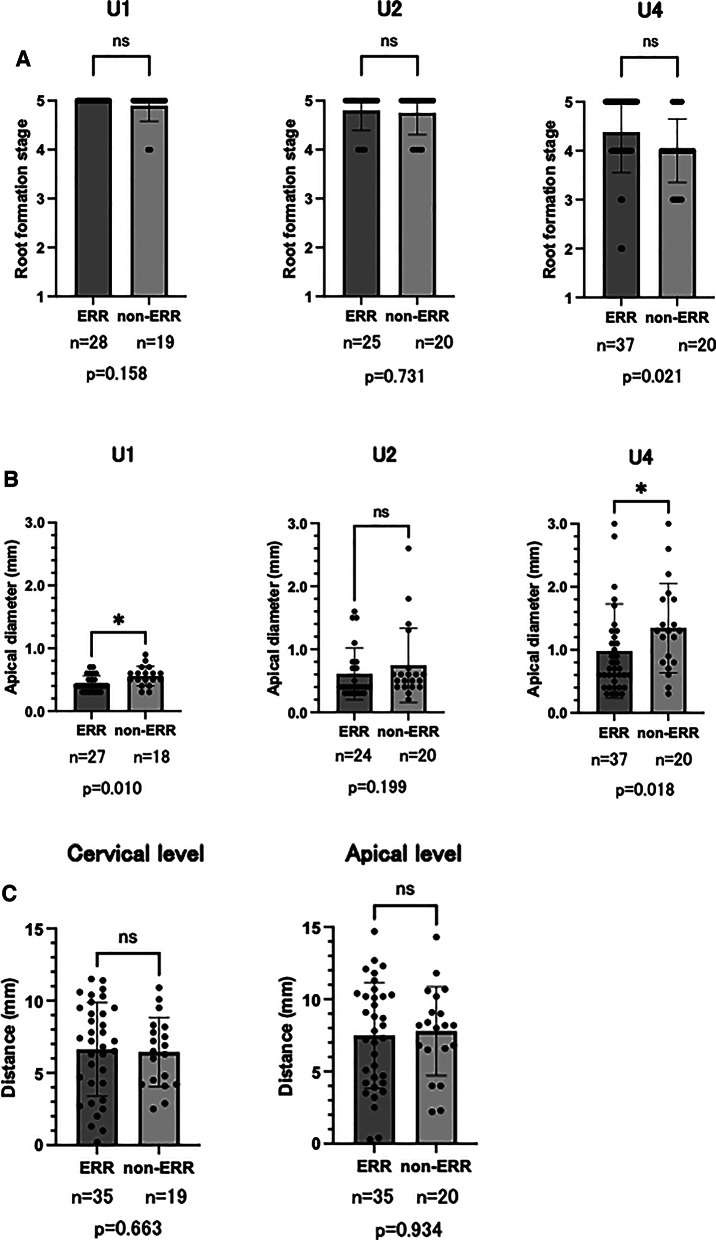
Comparison of developmental and spatial variables according to the presence of external root resorption. **A** Root formation stage; **B** AFD; **C** U2-U4 distance. * : p < 0.05, * * < 0.01. Abbreviations: ERR, external root resorption; AFD, apical foramen diameter; U1, maxillary central incisor; U2, maxillary lateral incisor; U4, maxillary first premolar

In exploratory tooth-level comparisons, the AFDs of U1 and U4 were smaller in the ERR group. Regarding root formation stages, U4 was initially more advanced in the ERR group than in the non-ERR group (unadjusted *p* = 0.021), whereas no clear differences were observed for U1 or U2 (unadjusted *p* = 0.158 and *p* = 0.731, respectively) (Fig. 4A). However, the difference in root formation stage of U4 was no longer statistically significant after Holm correction (adjusted *p* = 0.061). AFDs of U1 and U4 were smaller in the ERR group (adjusted *p* = 0.030 and *p* = 0.036, respectively), while no clear difference was observed for U2 (adjusted *p* = 0.199) (Fig. 4B). No clear differences were observed in the inter-root distance between U2 and U4 at either the apical or cervical levels (unadjusted *p* = 0.934 and *p* = 0.663, respectively) (Fig. 4C).

### Other analyses (confounded adjusted and subgroup analyses)

As an exploratory supplementary analysis, affected-side-level mixed-effects logistic regression models were fitted to account for clustering within patients. The model including minimum root formation stage did not provide stable estimates, as the final Hessian matrix was not positive definite and the standard errors were extremely large. Therefore, this model was not considered suitable for inference. The model including maximum AFD yielded interpretable estimates, but maximum AFD was not significantly associated with side-level ERR occurrence (OR = 2.44, 95% CI: 0.72–8.31, *p* = 0.150). The random intercept variance for patient ID was significant (variance = 6.55, 95% CI: 2.95–14.54, *p* = 0.014), supporting the presence of within-patient clustering and the need to account for clustering in this dataset.

## Discussion

### Main findings in the context of the existing evidence, interpretation

With the increasing use of CBCT in recent years, accurate 3D evaluation of IMCs has become increasingly feasible [14, 17, 18]. The present study utilized CBCT images to investigate risk factors for ERR of adjacent teeth, particularly focusing on the potential influence of the root formation stage of the adjacent teeth to the IMCs.

In the present study, ERR in teeth adjacent to IMCs was observed in 72.5% of patients and 37.1% of all examined teeth. Among the specific tooth types, U2 exhibited the highest incidence (31.5%), followed by U1 and U4. This distribution likely reflects the anatomical relationship where the eruption pathway of the maxillary canine typically passes in close proximity to the root apex of the U2 [11]. The lack of a significant difference in ERR prevalence between unilateral and bilateral impactions suggests that the laterality alone was not strongly associated with ERR in this sample.

Notably, 82.8% of the IMCs in this Japanese sample were positioned buccally. This contrasts with reports from Caucasian populations [11], where palatal impaction is more predominant, but aligns with the characteristic trends observed in Asian populations [19]. Despite these population-specific differences in impaction patterns, the overall prevalence of ERR in this study was comparable to, or even higher than, that reported in other CBCT-based investigations [1]. These findings strongly support the established concept that physical contact between the IMC and the adjacent root plays a pivotal role in the pathogenesis of ERR, regardless of the buccolingual position of the impaction.

Within this spatial framework, developmental indicators such as root formation stage and AFD may add contextual information rather than replace established spatial predictors. Root formation stage and AFD were analyzed as separate variables because they represent different aspects of tooth development: root formation stage reflects the categorical progression of tooth maturation, whereas AFD provides a quantitative measure of apical morphology.

The smaller AFDs observed in U1 and U4 within the ERR group suggest that root maturity may be related to resorption susceptibility. However, the supplementary affected-side-level mixed-effects analysis did not demonstrate a statistically significant independent association between maximum AFD and ERR occurrence. Therefore, AFD should be interpreted as a potential developmental indicator that may be clinically informative when considered together with spatial findings, rather than as an independent predictive marker. Furthermore, the present study evaluated macroscopic morphology and did not directly assess cellular, histologic, or biochemical mechanisms. Therefore, the observed association between smaller AFD and ERR should not be interpreted as direct evidence of reduced reparative capacity or any other biological mechanism [20, 21].

The affected-side-level mixed-effects analysis did not demonstrate a statistically significant independent association between maximum AFD and side-level ERR occurrence. This finding should not be interpreted as excluding a potential role of developmental status, because the analysis was exploratory and constrained by the retrospective sample size and by outcome-related missingness of developmental measurements. In particular, teeth with severe apical resorption may lack measurable anatomical landmarks for root formation stage or AFD, and these teeth are clinically important. Therefore, tooth-level predictive modeling based only on measurable developmental variables could underestimate or distort the association between developmental status and ERR.

Direct physical proximity between the IMC and adjacent roots is already a well-established primary predictor of ERR [1, 5, 16, 17]. The U2-U4 distance was therefore not intended to compete with, or replace, direct canine-root distance. Rather, it was selected to describe the available inter-root eruption corridor and the severity of teeth crowding. Direct canine-root distance is clinically intuitive, but in cross-sectional CBCT data it may partly represent the current contact or resorption state. In this study, no clear difference in U2-U4 distance was observed between ERR and non-ERR groups, suggesting that available space alone should not be used as a stand-alone risk marker.

In the present study, no clear association was observed between the buccolingual position of the IMC and the occurrence of ERR. This finding should be interpreted cautiously because the palatal subgroup was small, and previous CBCT studies have suggested that palatally impacted canines may have shorter distances to adjacent teeth and greater spatial constraints [2, 22]. Thus, our results should not be interpreted as contradicting the importance of spatial proximity. Rather, they indicate that buccolingual classification alone was not a sufficient discriminator of ERR in this sample.

The integrated evaluation of spatial and developmental factors may support a more comprehensive clinical assessment of IMC cases in preadolescent patients. Because irreversible ERR of the anterior teeth can affect oral function and psychosocial development, early identification of patients who may require detailed imaging or timely intervention is clinically important. In practice, assessment of adjacent root formation on panoramic radiographs may help determine the appropriate timing for detailed CBCT evaluation, while respecting the ALARA principle. These findings should be regarded as hypothesis-generating and should be validated in larger longitudinal studies.

A methodological strength of this study was the standardized MPR-based measurement protocol and high intra-examiner reproducibility, including an ICC of 0.95 for AFD. However, measurement reliability should not be equated with biological validity or predictive performance. The CBCT definition of ERR was designed to distinguish external root-surface defects from normal root development, but this distinction can remain challenging in developing teeth and warrants prospective validation.

### Limitations

The present study has several limitations. First, because this is a single-center retrospective cross-sectional study, it cannot establish strict longitudinal causal relationships among the dynamic eruption process of the IMC, the maturation of adjacent teeth, the temporal progression of events such as primary tooth exfoliation, and the occurrence of ERR. Another important limitation is the potential outcome-related missingness of developmental measurements. In teeth with severe ERR involving the apical region, the original root formation stage and AFD may be unmeasurable because the anatomical landmarks required for these measurements have been resorbed. Excluding such teeth from tooth-level regression models could remove the most clinically relevant severe cases and introduce selection bias. For this reason, the present study primarily compared affected sides with and without ERR, and the mixed-effects logistic regression models were interpreted as exploratory supplementary analyses rather than definitive predictive models. Future studies should use prospective designs with predefined rules for handling severe resorption and unmeasurable apical morphology.

### Generalizability

Because the study sample was restricted to a specific Japanese population and CBCT was performed only when further detailed assessment was clinically necessary, caution should be exercised when generalizing the results. Racial background, skeletal patterns, occlusal patterns, and growth characteristics may influence impaction trends and ERR risk [19]. Therefore, these findings cannot be directly extrapolated to all populations. Multicenter, multi-population studies with larger samples and longitudinal designs are required to clarify whether developmental indicators add clinically useful information beyond established spatial predictors and to evaluate their clinical utility in risk assessment.

## Conclusions

The findings suggest that developmental indicators, including AFD, may provide clinically relevant information when assessing ERR associated with IMCs. Developmental status should be interpreted as a potential modifying factor in conjunction with established spatial relationships, rather than as an independent predictive marker. Further prospective studies with larger samples and predefined strategies for handling severe resorption are needed.

## Data Availability

The datasets used and/or analyzed during the current study are available from the corresponding author on reasonable request.
